# Diagnostic accuracy of multi-slice computed tomography in children with Abernethy malformation

**DOI:** 10.1186/s12880-019-0396-3

**Published:** 2019-12-17

**Authors:** Chen Guo, Yu-Min Zhong, Qian Wang, Li-Wei Hu, Xiao-Hong Gu, Hong Shao, Wei Wu, Jian-Feng Cao, Hai-Sheng Qiu

**Affiliations:** Diagnostic Imaging Center, Shanghai Children’s Medical Center affiliated with Shanghai Jiao Tong University School of Medicine, 1678 Dong Fang Road, Shanghai, 200127 People’s Republic of China

**Keywords:** Abernethy malformation, Angiography, Tomography

## Abstract

**Background:**

Abernethy malformation is a rare congenital abnormality. Imaging examination is an important method for the diagnosis. The purpose of this study was to demonstrate manifestations of multi-slice computed tomography (MSCT) in Abernethy malformation and its diagnostic accuracy.

**Methods:**

Fourteen children with Abernethy malformation were admitted to our center in China between July 2011 and January 2018. All 14 patients (eight males and six females) received MSCT and digital subtraction angiography (DSA) while eight patients also received ultrasound. The patients’ age ranged from 1 to 14 (median age 8 years old). The clinical records of the patients were retrospectively reviewed. MSCT raw data were transferred to an Advantage Windows 4.2 or 4.6 workstation (General Electric Medical Systems, Waukesha, WI). We compared the findings of MSCT with DSA and surgical results in order to ascertain diagnostic accuracy.

**Results:**

Three cases had type Ib Abernethy malformation and eleven cases had type II. Two cases of type II Abernethy malformation were misdiagnosed as type Ib using MSCT. Comparing the findings of MSCT with DSA and surgical results, the accuracy of MSCT was 85.7% (12/14), in which 100.0% (3/3) were type Ib and 81.8% (9/11) were type II. Clinical information included congenital heart disease, pulmonary hypertension, diffuse pulmonary arteriovenous fistula, abnormal liver function, hepatic nodules, elevated blood ammonia, and hepatic encephalopathy. Eleven cases were treated after diagnosis. One patient with Abernethy malformation type Ib (1/3) underwent liver transplantation. Seven patients with Abernethy malformation type II (7/11) were treated by shunt occlusion, received laparoscopy, or were treated with open surgical ligation. Another three patients (3/11) with Abernethy malformation type II were treated by interventional portocaval shunt occlusion under DSA.

**Conclusion:**

MSCT attains excellent capability in diagnosing type II Abernethy malformation and further shows the location of the portocaval shunt. DSA can help when it is hard to determine diagnosis between Abernethy type Ib and II using MSCT.

## Background

Abernethy malformation is a rare congenital abnormality that was described by John Abernethy in 1793. It is characterized by a congenital extrahepatic portosystemic shunt between the portal vein and systemic circulation. It was defined by Morgan et al. [1] in 1994 as type Ia, type Ib, and type II, based on whether the liver is perfused with portal blood, and whether the superior mesenteric vein and splenic vein join to form a confluence in the portal vein. A variety of clinical presentations can be evidenced in those with Abernethy malformation. It is very difficult to diagnose solely on the basis of clinical manifestations. Imaging examination is an important method for the diagnosis of Abernethy malformation. Conventional cine angiography, which is considered a gold standard, requires catheterization with ionizing radiation. Recent developments in CT technology with better spatial and temporal resolution have increased its clinical use in children. The source images can be reformatted in any desired plane for better visualization of the vascular anatomy. The aim of this study was to demonstrate manifestations of MSCT in Abernethy malformation and to evaluate its diagnostic accuracy.

### Types of Abernethy malformation

**Fig. 1:**
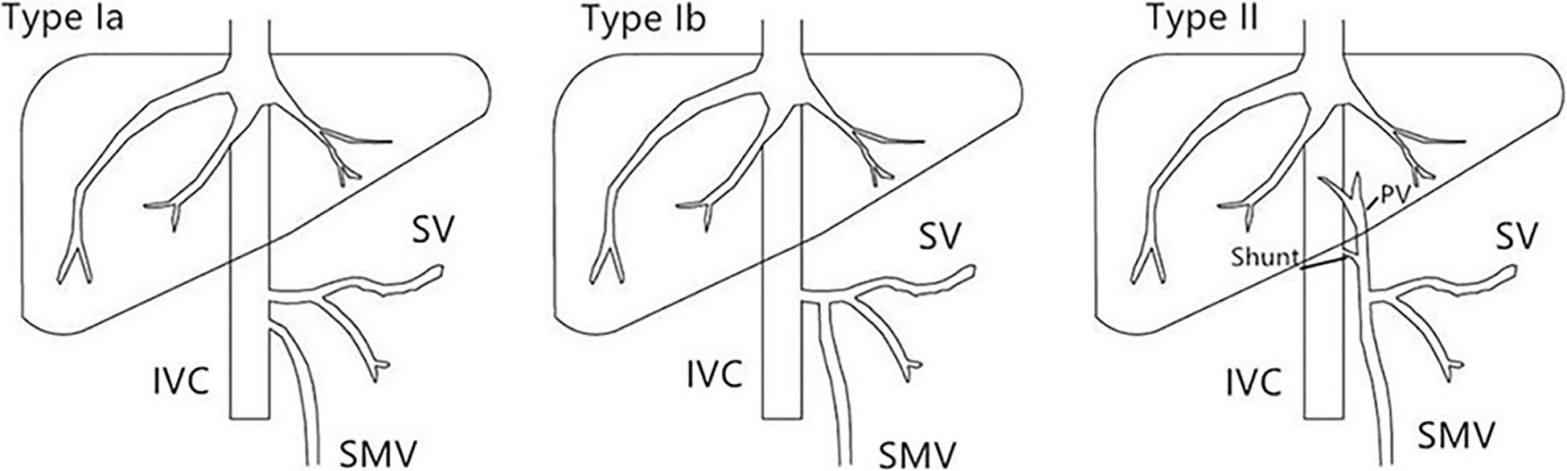
Classification of Abernethy Malformation

There are two types of Abernethy Malformation (Fig. 1). Type I is defined as a complete porto- systemic shunt, in which the portal vein merges with the inferior vena cava (IVC) in an end-to-end shunt. In this situation, the liver is not perfused with portal blood. Type I is further sub-classified into types Ia and Ib according to whether superior mesenteric vein and splenic vein join to form a confluence with the portal vein or not. Abernethy Malformation type II refers to a partial shunt, consisting of a side-to-side connection between the portal vein and the systemic venous circulation, with partial portal blood flow to the liver.

## Methods

### Patients

The ethics commission of our hospital approved this retrospective study. Fourteen pediatric cases of Abernethy malformation were admitted to our hospital between July 2011 and January 2018. All 14 patients (eight males and six females) received MSCT and DSA (digital subtraction angiography). The age of all patients ranged from 1 to 14 years, with a median age of 8 years. The clinical records of the patients were reviewed. All patients underwent DSA under anesthesia. For MSCT examination, five patients younger than 6 years of age were sedated with orally administered chloral hydrate or/and intramuscular phenobarbital.

### CT scanning protocols

MSCT angiography was performed on a 16-row CT scanner (Lightspeed 16, General Electric Medical Systems, Milwaukee, WI, USA)(case 1–11) or a 64-slice high definition CT scanner (Discovery CT 750 HD, General Electric Medical Systems, Waukesha, WI, USA)(case 12–14). The parameters for CT using the 64-slice CT scanner were as follows: a low-dose CT protocol (100–120 kVp, 50 mA) and the following parameters: collimation 16 × 1.25 mm, pitch 1.375:1, table feed 13.75 mm/rotation, slice thickness 0.625 mm or 1.25 mm. The parameters for CT using 16-slice CT scanner were as follows: 120 kV, 100 mA, pitch 1.375:1, table feed 13.75 mm/rotation, slice thickness 0.625 mm. Nonionic contrast agent (2 ml/kg) was injected throughout the scan via a peripheral vein by a pressure injector. All patients underwent arterial and portal phase scans.

The volumetric CT dose index (CTDI vol) and dose length product (DLP) were recorded. The effective dose (ED) (mSv) was derived from the dose-length product and age-dependent conversion coefficients.

### CT image processing

CT raw data were transferred to an Advantage Windows 4.2/4.6 workstation (General Electric Medical Systems). Maximum intensity projection (MIP) and multiplanar reformation (MPR) were the primary methods for visualization and evaluation.

### DSA

DSA was performed using a digital subtraction angiography machine (Allura Xper FD10/10, PHILIPS, the Netherlands) or (INOVA 2100, General Electric Medical Systems, France) in all patients. After the puncture of arteries and veins from inguinal region, the catheter was placed at the shunt for angiography and pressure gradients were recorded. MSCT findings were compared with that of DSA.

### Statistical analysis

The same two radiologists with more than 10 years experiences also confirmed CT diagnosis based on the CT findings separately. Any discrepancies were resolved by independent review. By comparing the findings of MSCT with DSA, we were able to calculate diagnostic accuracy.

## Results

### Patient information

Fourteen cases were admitted to our hospital (Table 1). The fourteen patients experienced a variety of clinical features, which included hematochezia (case 3), cyanosis (cases 4,9,14), fatigue (cases 5,7,8,12), hematuria (case 10), and pulmonary hypertension (cases 2,3,6,7,11,12,13). Six patients (6/14) were diagnosed as having congenital heart disease (ventricular septal defect (VSD), patent ductus arteriosus (PDA), atrial septal defect (ASD), polysplenia syndrome, and partial anomalous pulmonary venous connection (PAPVC)). Two (2/14) had abnormal liver function, ten (10/14) had hyperammonemia, three (3/14) had nodular liver lesions, one patient (1/14) had hepatic encephalopathy, and four (4/14) patients had pulmonary arteriovenous fistula.

**Table 1:** Summary of findings in patients with Abernethy Malformation

Case No.	Age (y)/Sex	Clinical Features	Fistula classification and anatomy	Treatment
1	11 years/Female	VSD, Hyperammonemia, Abnormal liver function	Type Ib(PV-IVC)	Lost to follow-up
2	6 years/Female	PH, Nodular liver lesions	Type Ib(PV-IVC)	Liver transplantation
3	12 years/Male	Hematochezia, PH, Hyperammonemia	Type Ib(SV-iliac vein-IVC)	Lost to follow-up
4	11 years/Female	Cyanosis, Pulmonary arteriovenous fistula, Hyperammonemia	Type II(PV-IVC)	Surgical shunt occlusion
5	9 years/Male	Fatigue, PDA, Nodular liver lesions	Type II(PV-IVC)	Surgical shunt occlusion
6	4 years/Male	PH, Hyperammonemia	Type II(PV-IVC)	Surgical shunt occlusion
7	9 years/Male	Fatigue, PH, Hepatic encephalopathy, Heart insufficiency, Hyperammonemia	Type II(PV-IVC)	Surgical shunt occlusion
8	14 years/Male	Fatigue, VSD, Hyperammonemia, Nodular liver lesions	Type II(PV-IVC)	Lost to follow-up
9	5 years/Male	Cyanosis, Pulmonary arteriovenous fistula, Hyperammonemia	Type II(PV-IVC)	Interventional portocaval shunt occlusion under DSA
10	10 years/Female	Hematuria, Renal vascular malformation, Hyperammonemia	Type II(SV-iliac vein-IVC)	Surgical shunt occlusion
11	3 years/Female	ASD,PH, Abnormal liver function	Type II(PV-IVC)	Interventional portocaval shunt occlusion under DSA
12	4 years/Male	Fatigue, ASD/PAPVC, Pulmonary arteriovenous fistula, Hyperammonemia, PH	Type II(PV-LRV-IVC)	Surgical shunt occlusion
13	7 years/Male	PH, Hyperammonemia	Type II(PV-IVC)	Surgical shunt occlusion
14	1 year/Female	Cyanosis, ASD, Polysplenia syndrome, Pulmonary arteriovenous fistula	Type II(SMV-LRV-IVC)	Interventional portocaval shunt occlusion under DSA

*PV* = portal vein; *IVC* = inferior vena cava; *SMV* = Superior mesenteric vein; *LRV* = left renal vein; *ASD* = atrial septal defect; *VSD* = ventricular septal defect; *PDA* = patent ductus arteriosus; *PAPVC* = partial anomalous pulmonary venous connection; *PH* = pulmonary hypertension

### MSCT findings

**Fig. 2:**
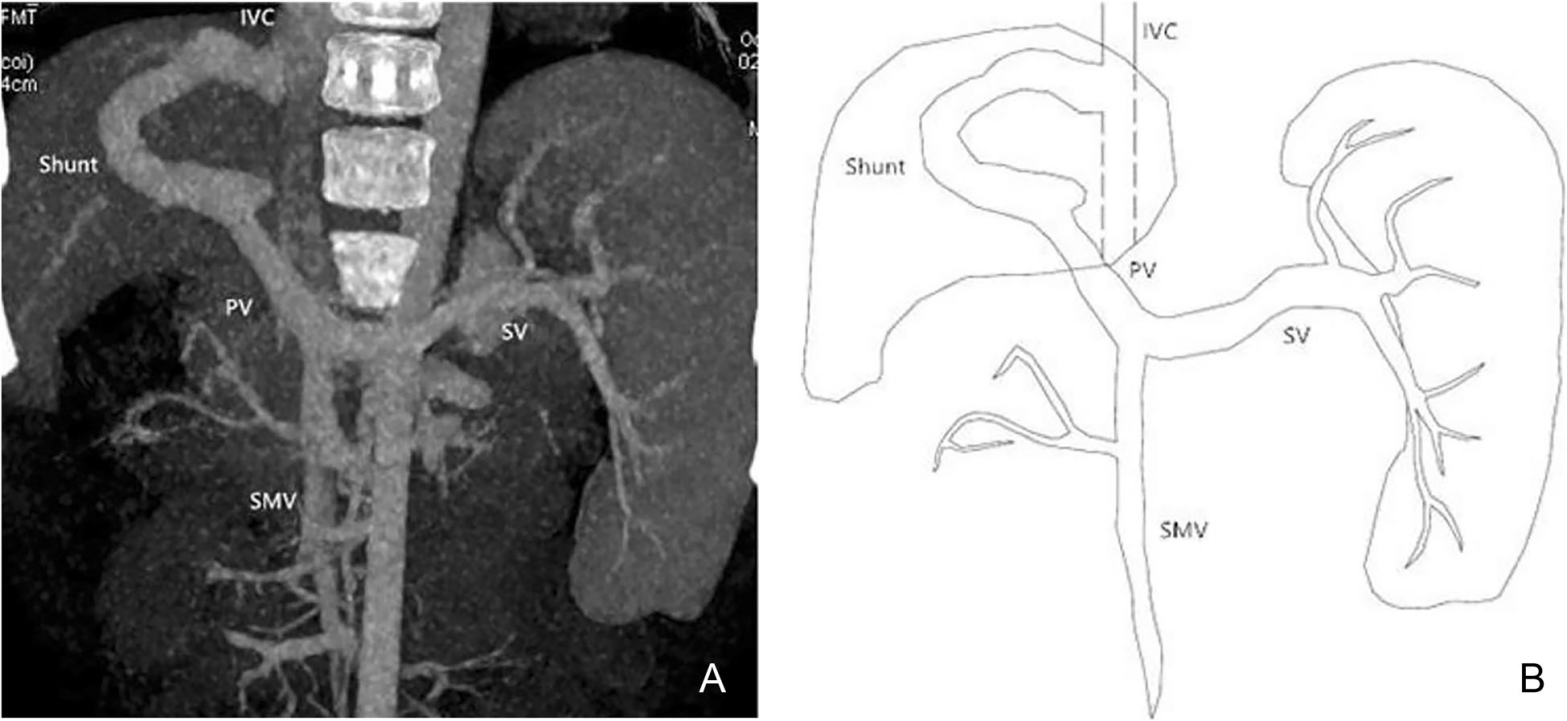
Male, 7 years old, Abernethy II. Abdominal CT angiography maximum intensity projection (MIP) image (portal phase) shows the shunt of extrahepatic portosystemic between portal vein and inferior vena cava

**Fig. 3:**
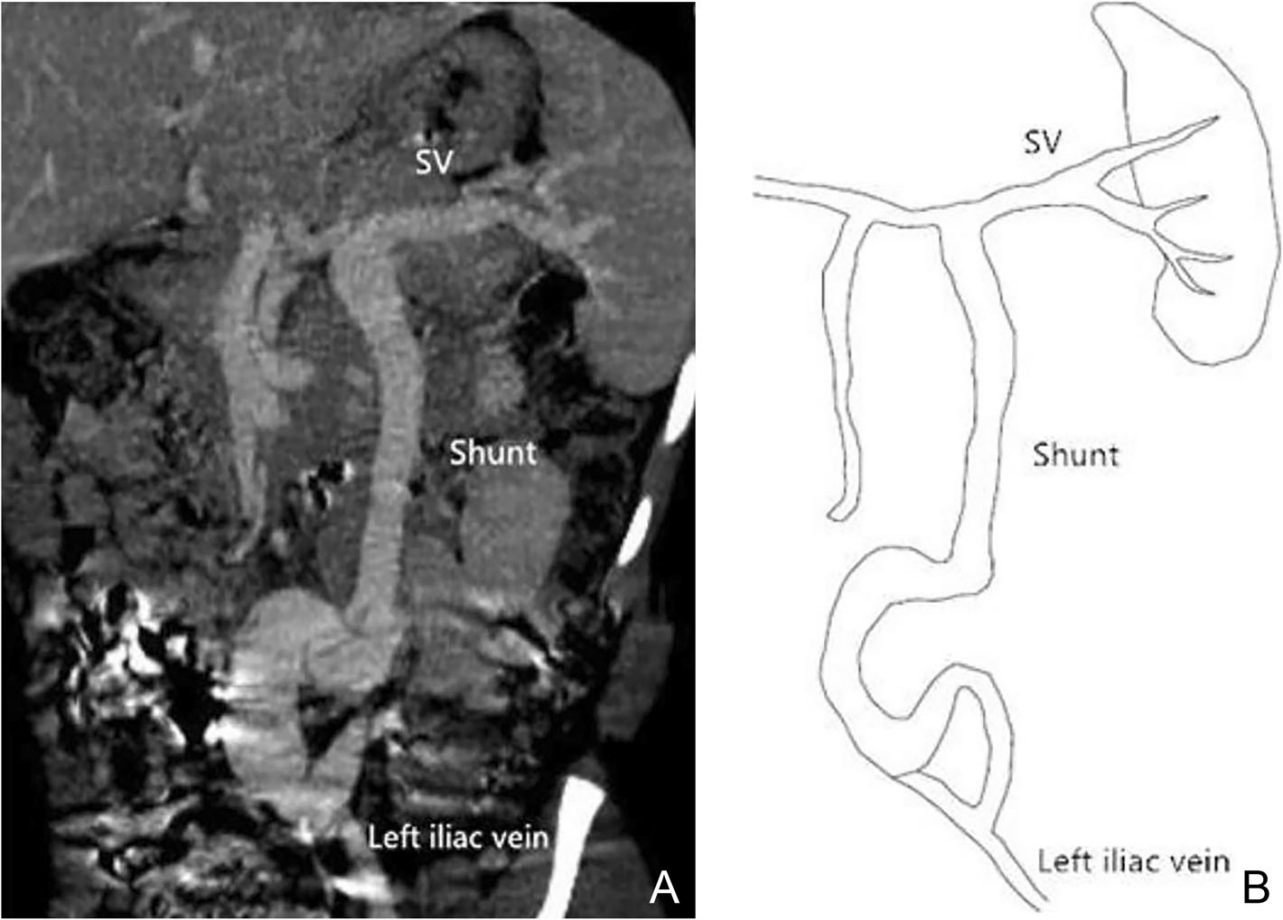
Female, 10 years old, Abernethy II. Abdominal CT angiography maximum intensity projection (MIP) image (portal phase) shows the shunt of extrahepatic portosystemic between splenic vein and left iliac vein

Using MSCT, we were able to show the location of the shunt between the portal vein and systemic circulation for all 14 cases. Among the fourteen patients, ten presented with single vessel shunt between portal vein and IVC (Fig. 2), one with shunt between the portal vein and left renal vein, one with shunt between the superior mesenteric vein and left renal vein, one with shunt between the splenic vein and right iliac vein, and one with shunt between splenic vein and the left iliac vein (Fig. 3).

The two radiologists’ diagnosis was the same. Five children with Abernethy malformation type Ib and nine children with type II were identified by MSCT. Compared with DSA and surgical results, two cases of type II Abernethy malformation were misdiagnosed as type Ib Abernethy malformation in MSCT (Table 2). The accuracy rate of this method was 85.7% (12/14). Intrahepatic lesions were found in three patients using MSCT.

**Table 2:** Accuracy of Classification of Abernethy malformation in MSCT Compared with DSA

DSA	MSCT	Accuracy (%)
	Ia Ib II	
Ia(*n* = 0)	0 0 0	/
Ib(*n* = 3)	0 3 0	100.0
II(*n* = 11)	0 2 9	81.8

The mean DLP (Dose length product) for cases using the 16-row CT scanner was 371.353 ± 176.583 mGy-cm, mean CTDI was 14.49 ± 5.973 mGy, resulting in an estimated mean effective dose (ED) of 6.948 ± 4.561 mSv. The DLP, CTDI and calculated ED for cases using the 64-slice high definition CT scanner were 106.100 ± 48.312 mGy-cm, 4.50 ± 2.155 mGy and 2.593 ± 1.490 mSv.

### DSA findings

All patients who received DSA had their shunt identified by DSA. Hypoplastic portal veins were visualized in patients of which two were not visualized in MSCT.

### Surgical

Eleven cases were treated after diagnosis. One patient with Abernethy malformation type Ib (1/3) underwent liver transplantation. Seven patients with Abernethy malformation type II (7/11) were treated by shunt occlusion, received laparoscopy, or were treated with open surgical ligation. Another three patients (3/11) with Abernethy malformation type II were treated by interventional portocaval shunt occlusion under DSA. The remaining 3 cases (two with type Ib and one with type II) were lost to follow-up.

Of the eleven treated patients, eight were followed up after treatment (case 2, 5, 6, 7, 9, 12, 13, 14). Their blood oxygen saturation reached normal level and their symptoms were alleviated to varying degrees compared with those before operation. Among the eight cases, five patients’ serum ammonia level returned to normal (case 6, 7, 9, 12, 13, preoperative serum ammonia level was normal in other three patients.). DSA was reexamined in four patients within 1–3 years after treatment, which showed that the development of intrahepatic portal vein was better than that before treatment (case 5, 6, 7, 14). Pulmonary arteriovenous fistula disappeared in two patients (case 9, 14). Pulmonary hypertension still existed in three patients (case 7, 12, 13).

## Discussion

Abernethy malformation, also known as congenital extrahepatic portosystemic shunt, is an extremely rare syndrome. The majority of affected patients described to date were under the age of 18 [2–4]. Type I malformations have complete portosystemic shunts that do not perfuse the liver and are predominantly found in females [5–7]. Type II malformations have a hypoplastic portal vein leading to liver perfusion.

As shown in our cases, Abernethy malformations have a wide spectrum of symptoms including hematochezia, cyanosis, fatigue, hematuria, and others. Other relevant symptoms recorded in studies include nausea, vomiting, epigastric pain, asthenia, anorexia, jaundice, and dyspnea [4]. The symptoms may be mild and non-specific, with some patients being asymptomatic [8, 9]. Pulmonary hypertension is a frequent initial clinical manifestation of the disease and hyperammonemia is a common manifestation. In our group, 50% (7/14) patients presented with unexplained pulmonary hypertension, and 71.4% (10/14) had hyperammonemia. Severe elevation of blood ammonia can lead to hepatic encephalopathy but only one of our 14 cases had this manifestation.

Abernethy malformation is frequently associated with cardiovascular abnormalities, including atrial septal defect (ASD), patent foramen ovale (PFO), ventricular septal defect (VSD), patent ductus arteriosus (PDA), tetralogy of Fallot (TOF) and dextrocardia [1, 5, 6, 10, 11]. In our 14 cases, 42.9% (6/14) cases had a history of congenital heart disease, and included ASD, VSD, PDA, PAPVC, and polysplenia syndrome (one patient with type I and five patients with type II). Some studies have indicated that type I Abernethy malformation occurs more frequently in girls and is associated with other congenital anomalies, while type II Abernethy shunts are rarely seen with these malformations [9, 12, 13]. Two of three cases of type I Abernethy malformation were female patients. However, in our eleven cases of type II, five cases had congenital heart disease. Nodular liver lesions were observed in almost half of the reported cases, and attributed to the absence of portal blood flow and compensatory increased hepatic arterial blood flow. The most frequently observed lesion is focal nodular hyperplasia, while others include nodular regenerated hyperplasia, hepatoblastoma, hepatic adenoma, hepatocellular carcinoma, and cirrhosis [4]. Only 21.4% of our cases (3/14) showed nodular liver lesions but were not confirmed by pathology.

When a portosystemic shunt carries an increased risk of hepatic encephalopathy or is associated with the development of liver tumors, it requires treatment [14]. Early diagnosis is important. Many image modalities can be used for diagnosing Abernethy malformation, including ultrasound, CT, DSA, and MRI.

Ultrasound is a non-invasive method of examination. Eight of the fourteen cases underwent US in our study, and all eight cases were able to confirm Abernethy malformation by showing the exact position of the shunt. However, compared with MSCT, US did not clearly show the development of the intrahepatic portal vein. Hypoplastic portal veins were found in only two cases using US. It would appear that confirmation of the type of Abernethy malformation by US is less efficient compared with MSCT.

**Fig. 4:**
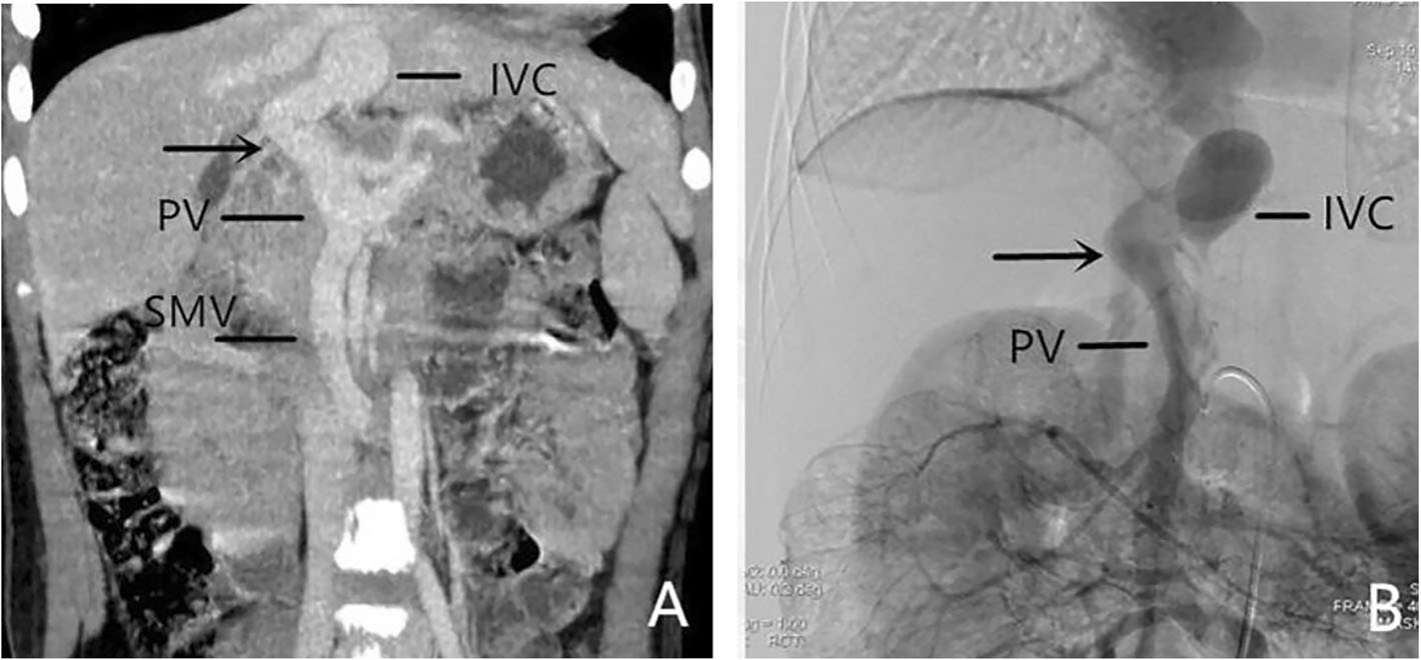
Female, 11 years old, Abernethy Ib. **a**: Abdominal CT angiography maximum intensity projection (MIP) image (portal phase) shows the presence of extrahepatic portosystemic shunt (arrow) with absence of intrahepatic portal veins. **b:** DSA image confirmed the MSCT result

MSCT is a non-invasive imaging technique with high spatial resolution and the ability to rapidly test for the diagnosis of Abernethy malformation. We have a low-dose CT protocol (100–120 kVp, 50 mA) for children since the procedure involves ionizing radiation. In our 14 cases, MSCT was able to show the exact location of the portocaval shunt (Fig. 4) in all cases. Through post-processing technology (MPR, MIP, VR), CT can visually display the location of shunt and its relationship with surrounding tissues. Among these imaging examinations, MSCT is a relatively efficient method. The major advantages of CTA are rapid examination and easy availability, with a very short scanning time [15]. First of all, it is a non-invasive and rapid method of imaging examination. Secondly, CT can accurately display the relationship between the location of shunt and the surrounding tissues. MSCT can give accurate classification of Abernethy malformation (Type II) especially in cases where the intrahepatic portal vein can be accurately demonstrated. Therefore, MSCT can accurately follow up these patients without using DSA each time.

Radiation dose is the main concern in imaging pediatric patients. The organ doses delivered from a traditional CT examination result in a potentially increased risk of radiation-induced carcinogenesis, particularly for children [16]. Of our 14 cases, three cases used 64-slice high definition CT scanner. The DLP, CTDI and calculated ED for cases using the 64-slice high definition CT scanner were significantly lower than those using 16-row CT scanner. However, advances in CT technology can reduce the radiation dose to patients on the premise of ensuring image quality. GE Healthcare developed a novel iterative reconstruction technique, ASiR-V, which uses almost full iterative reconstruction system with the potential for significant radiation dose reduction and superior image quality than conventional ASiR and a shorter imaging processing time than the model-based iterative reconstruction [17]. The use of wide detector technology and the acceleration of scanning speed also help to reduce the radiation dose of children’s CT examination [18].

The conventional original non-contrast MRA techniques such as time of fight and phase contrast MR angiography produce static images with prolonged acquisition time and cannot detect small vessels. Several non-contrast and contrast enhanced MR angiography (MRA) techniques have been developed [19]. New developments of non-contrast MRA include cardiac-gated 3D fast-spin-echo, arterial spin labeling, and balanced steady-state free-precession. These techniques have better image quality and shorter examination time, but they may be associated with flow artifacts. Due to problems such as sedation, lower spatial resolution, and long examination time, none of our cases underwent MRI examination. For young children, longer examination time means deeper levels of sedation. The number of procedures requiring anesthesia and or sedation in these children is growing and the cumulative effect of these repeated exposures on the developing brain is unknown [20]. The possible side effects of sedatives on children, especially newborns, cannot be ignored. Recent several vivo studies have shown that early use of anesthetics and sedatives can lead to permanent structural and functional changes of central nervous system (CNS) [21]. Compared with MRI, CT has a very fast scanning speed. Wide-detector CT makes CT scanning with lighter to no sedation possible due to the advancement of CT technology [20]. It is believed that with the advancement of technology, the acceleration of scanning speed and the optimization of image quality, MRA will play an increasingly important role in the diagnosis of small vessel diseases in children.

**Fig. 5:**
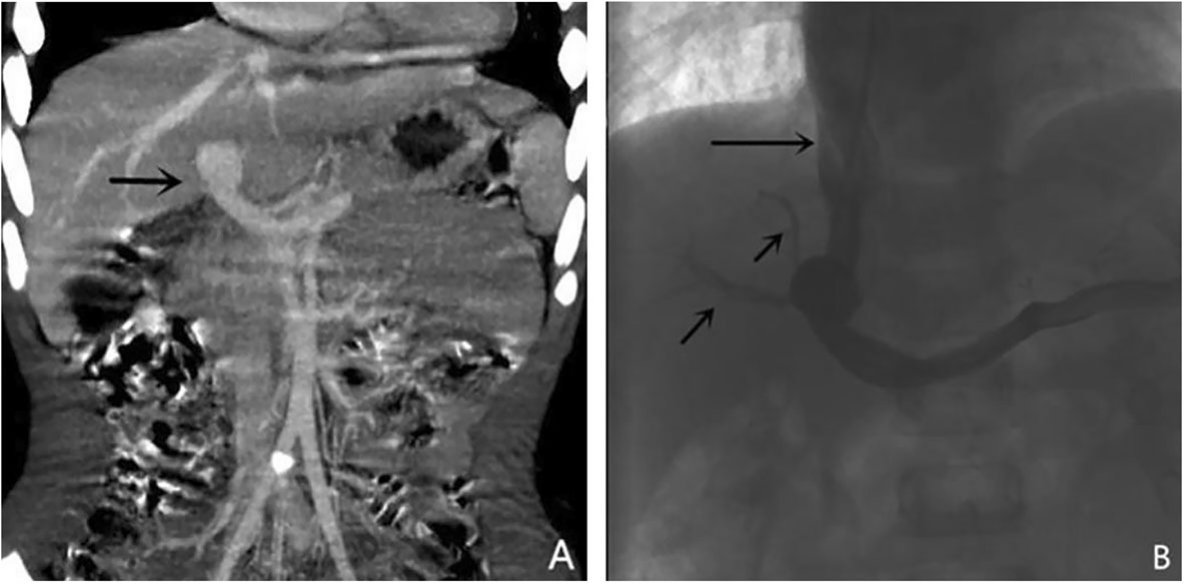
Male, 3 years old, Abernethy II. **a:** Abdominal CT angiography maximum intensity projection (MIP) image (portal phase) shows the presence of extrahepatic portosystemic shunt (arrow) with absence of intrahepatic portal veins and diagnoses Abernethy Ib;**b:** DSA image shows the shunt (long arrow) and the tiny intrahepatic portal veins (short arrow)

However, some patients with Abernethy type II have extremely hypoplastic portal veins distal to the shunt that are sometimes difficult to visualize with CT angiography scan. In these patients, DSA is still essential for studying the anatomy of the shunt. Among our fourteen cases, two cases of type II Abernethy malformation were misdiagnosed as type Ib Abernethy malformation using MSCT. As shown in Fig. 5, MSCT was not able to show the tiny intrahepatic portal veins, which could be seen in the DSA image. In the cases of Abernethy malformation, due to the presence of portosystemic shunt, the pressure of intrahepatic portal vein is higher than that of systemic vein. Contrast agents are more likely to enter the systemic vein through communicating branches. During DSA examination, after balloon occlusion in the shunt, the contrast agents failed to enter the systemic vein, which increased the pressure of the small portal vein branches in the liver [12]. Therefore, DSA images can show extremely hypoplastic portal vein branches in the liver which not able to be shown in CT images.

DSA remains the gold standard for the diagnosis of the disease, although it is an invasive imaging examination. However, it is especially useful for cases where the development of intrahepatic portal vein cannot be accurately demonstrated in MSCT, and consequently DSA is a necessary diagnostic regimen. Another important point is that some cases of Abernethy Malformation can be treated using DSA.

The treatment of Abernethy malformation is dictated by the classification. Type I malformations are definitively treated by liver transplantation due to complete absence of intrahepatic portal veins [22]. Type II malformations can be treated with either surgical ligation or endovascular shunt occlusion. When the shunt is not amenable to surgical closure, the only alternative is liver transplantation [13].

## Limitation

There are some limitations in our study. First of all, our research was a retrospective small sample-size study although given the rarity of the condition, we are restricted by the rare incidence of this condition. Secondly, the imaging examination and description of some of the cases were incomplete. Thirdly, some cases were lost to follow-up. In future, we will expand our sample size when performing a future study.

## Conclusion

In summary, it is essential to confirm the type of Abernethy malformation in the affected individual since there are different treatments for the two types. MSCT can accurately diagnose type II Abernethy malformation while showing the exact location of the portocaval shunt. Sometimes, it is hard to confirm the diagnosis between Abernethy type Ib and II because of the failure to visualize the extremely hypoplastic portal veins distal to the shunt with CT angiography. DSA still remains the gold standard in diagnosing Abernethy malformation.

## Abbreviations

ASD: Atrial septal defect
DSA: Digital subtraction angiography
IVC: Inferior vena cava
MIP: Maximum intensity projection
MPR: Multiplanar reformation
MSCT: Multi-slice computed tomography
PAPVC: Partial anomalous pulmonary venous connection
PDA: Patent ductus arteriosus
PFO: Patent foramen ovale
TOF: Tetralogy of Fallot
VSD: Ventricular septal defect
